# Sensorless Direct Field-Oriented Control of Induction Motor Drive Using Artificial Neural Network-Based Reactive Power MRAS

**DOI:** 10.3390/s25237135

**Published:** 2025-11-22

**Authors:** Marek Kubatko, David Bielesz, Stepan Kirschner, Kamal Hamani, Martin Kuchar, Tomas Mrovec, Michal Prazenica

**Affiliations:** 1 Department of Applied Electronics, Faculty of Electrical Engineering and Computer Science, VSB-Technical University of Ostrava, 708 00 Ostrava, Czech Republic; marek.kubatko@vsb.cz (M.K.); david.bielesz@vsb.cz (D.B.); stepan.kirschner@vsb.cz (S.K.); kamal.hamani.st@vsb.cz (K.H.); tomas.mrovec@vsb.cz (T.M.); 2 Department of Mechatronics and Electronics, Faculty of Electrical Engineering and Information Technology, University of Zilina, SK-010 26 Zilina, Slovakia; michal.prazenica@uniza.sk

**Keywords:** induction machine, sensorless control, MRAS, Q-MRAS, ANN-Q-MRAS, artificial neural network, DFOC

## Abstract

In this paper, advanced sensorless control methods of an induction machine (IM) with the use of a model reference adaptive system (MRAS) estimator are presented, specifically an MRAS using reactive power (Q-MRAS) with an implemented feedforward artificial neural network. Advantages of the proposed solution in comparison with the conventional Q-MRAS include better robustness, less dependence on the IM parameters, and stable operation in the regenerative mode. The simulations were performed in the MATLAB Simulink interface to validate the proposed approach. The algorithm was implemented in a single-core TMS320F28335 real-time Digital Signal Controller with a LabVIEW control panel. The experimental results were obtained on a three-phase experimental induction motor drive with a nominal power of 2.2 kW.

## 1. Introduction

The induction machine (IM) remains the most widely used type of electric motor in industry at present, due to its low maintenance costs, good mechanical (simple construction, absence of brushes or slip rings, in comparison with DC motors) and electrical properties, and relatively low manufacturing cost, especially in the case of squirrel-cage IMs. In terms of very high demands for dynamic states and the precision of measured values, there is a need for a sufficient control method of IMs.

Adopted approaches that meet the following requirements include field-oriented control or direct torque control. These methods are used to achieve AC drive control characteristics comparable to those of DC drives, which means independent control of the magnetic flux and torque of the machine. The high complexity of their control can be considered a disadvantage, which requires sufficient computational power, in comparison with DC drives. The reliability of the aforementioned control techniques also depends on rotor position data, which is typically obtained from a speed sensor with high resolution—often an incremental encoder, which is a mechanical component that is very sensitive and prone to failure [1]. Because of that, one of the core topics in the field of electric drives is controlling IMs without this sensor and evaluating the position of the rotor only using the DC-link voltage sensor and current sensors located in each phase of the motor. There are many variants of sensorless AC drives that have been proposed over the last few decades. Solutions differ in dynamics, accuracy, stability, computational demands, and mathematical methods. This approach is known as sensorless control of IMs [2,3,4,5,6,7,8,9,10,11].

Sensorless control of IMs can be broadly classified into two categories: methods that rely on the motor model, and those that do not. If the motor model is known, the speed can be determined from the measured values of the stator voltages and currents. Open-loop estimators use complete or partial equations of the machine model to estimate the rotor speed. The machine parameters are needed, and these methods are known for their poor stability at low speeds and a regenerative mode of operation, but today improvements are implemented in these methods to overcome the problematic areas, as mentioned in [7,8,12].

In methods with a motor model, extended-Kalman-filter-based algorithms and Luenberger observer speed estimation methods are included. These methods are robust to noise and are able to adapt to the observer in real time, leading to more precise estimation, but are more demanding in terms of computational power for the control system [9,10]. In addition, methods based on the injection of voltage or current signals, as well as increasingly popular soft computing approaches such as genetic algorithms [11] or control structures with artificial neural networks (ANNs), have been developed in recent years. Properly trained ANN-based methods can provide stable operation in various speed ranges. They are usually used in combination with other methods, such as estimators using model reference adaptive systems (MRASs) [13,14,15] or fuzzy logic systems [16].

**Figure 1:**
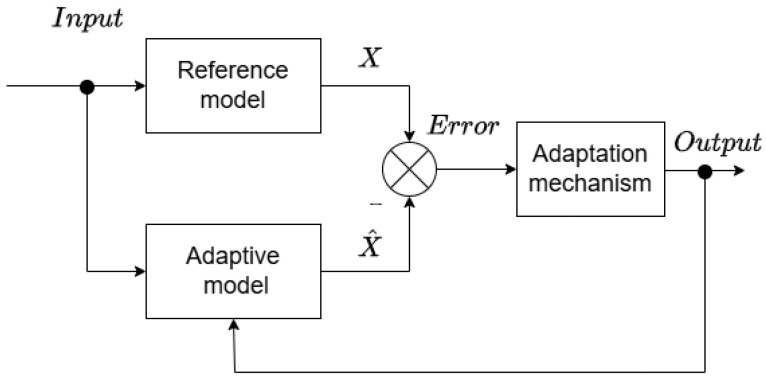
Block diagram of MRAS estimator.

MRAS structures are easy to implement because of their simplicity. The basic concept involves two estimators with an adaptation mechanism (see Figure 1). The first is a speed-independent reference model that generates an estimated state variable. The second is a speed-dependent adaptive model, whose output is compared with the output from the reference model within an adaptation algorithm. The result of this comparison is fed back into the input of the adaptive model as the estimated variable. The estimator output is adjusted to minimize the error between the reference and adaptive model outputs, ideally reaching zero.

Various MRAS observers exist, each differing in their structure, operating speed range, performance during dynamic and steady-state conditions, and state variables used for the speed estimation. All MRAS estimators and their accuracy are influenced by the IM parameter mismatch, because they are based on the mathematical model of an IM. Another common problem is the stability in the regenerative mode and in the low-speed regions [17,18]. According to the state variable, the MRAS estimators can be divided into the following groups:

- Rotor flux-based MRAS (RF-MRAS);
- Back electromotive force-based MRAS (BEMF-MRAS);
- Active or reactive power-based MRAS (P- or Q-MRAS);
- Virtual variable-based MRAS (X- and Y-MRAS);
- Current-based MRAS (CB-MRAS);
- Sliding-mode-based algorithm MRAS (SM-MRAS);
- Artificial-intelligence-based MRAS (ANN-MRAS);
- Fuzzy logic MRAS (FL-MRAS).

The RF-MRAS uses the voltage and current models of the IM for rotor flux evaluation. Issues associated with this type of estimator include poor stability in low-speed regions for motor and regenerative modes, high stator resistance dependency, and open integration included in the reference model for flux calculation [19,20].

Integration is not needed in the BEMF-MRAS type, but differentiation is included in the reference model, which can amplify noise in the system and make this estimator unstable in low-speed regions, as was the previous type of MRAS [21].

The conventional active and reactive power MRAS is an extended version of the BEMF-MRAS; its stability performance is the same as that of the previously mentioned method. The active power MRAS has overall stability problems. The reactive power MRAS (Q-MRAS) has better stability in the full operating range, and with the improvement made in [22], the authors removed the differentiation operation by calculating the reactive power directly in a synchronous reference frame. This approach improved the stability, but dependency on most parameters of the IM remained. The reactive power-based MRAS achieves better results than the RF- and BEMF-MRAS in the low speed range. However, this type of estimation has problems throughout the regenerative mode [22]. Therefore, improvements are needed for sufficient stability in the low speed range, and to make the system less dependent on changes in motor parameters.

The current-based MRAS uses an IM as a reference model, and there are two subtypes that differ in the usage of a voltage or current model for the rotor flux vector computations. The voltage rotor flux computation uses integration, and the current rotor flux computation has a problem with stability in the regenerative mode [17], but improvements have already been implemented and presented in [23]. According to the authors, a modified MRAS estimator is stable in the full operating range.

The next approach uses the so-called fictitious quantity in the X- and Y-MRAS estimators. They are dependent on the reference frame, and the rotor resistance and inductance parameter influence the accuracy of the speed estimation [24].

The last group implements neural networks or fuzzy algorithms in the MRAS structure or combines other approaches in the MRAS estimator, like sliding-mode observers. This group differs in its mathematical models of suitable state variables in both reference and adaptive models, and in the adaptation mechanism used for the rotor speed calculations. Fuzzy logic is focused on approximate reasoning, and the neural network approach has the advantage of learning the required algorithm without knowing the exact mathematical model of the system. Also, learning and improving during operation is available [17,25]. The reason for the implementation is the higher robustness of the system and the improvement in stability with the decrease in dependency on IM parameters.

### The Novelty of the Proposed Solution Is Summarized in the Following Points

**Figure 2:**
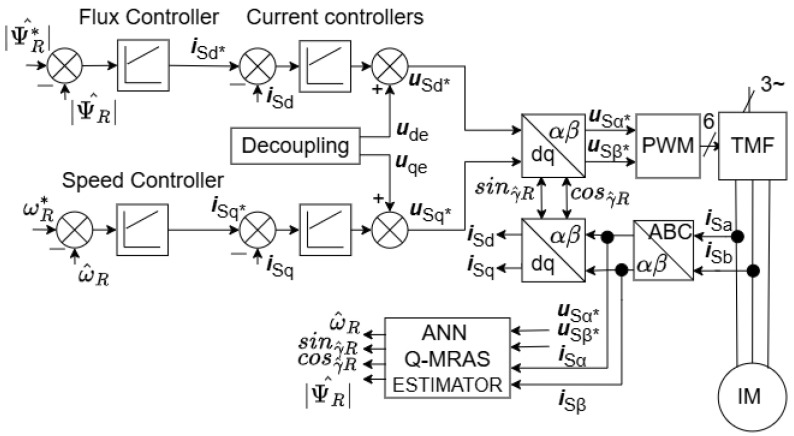
DFOC−based sensorless IM drive.

- In the approach, direct field-oriented control (DFOC) is used (see Figure 2) instead of indirect field-oriented control (IFOC), which is used for the Q-MRAS estimators presented in [22,25,26], and on which is this work based. The advantage is the closed-loop integration, which is more stable in comparison with IFOC. Moreover, in the DFOC structure, the rotor magnetic flux is directly available, and the flux PI controller is integrated, which is an advantage for flux-changing requests (fast initial excitation, field-weakening mode, etc.).
- The implemented artificial neural network used as the adaptive model in the proposed Q-MRAS solution brings better stability to the system and less dependency on motor parameters.
- The presented algorithm can be implemented in conventional Digital Signal Controllers without the need for expensive high-computing-power systems.
- Training data were obtained under no-load conditions, and the tested model was successfully generalized to the load condition of 20% due to the inclusion of dynamic transients in the training set. This training strategy significantly simplified both the data collection process and the drive setup.

A brief state of the art of the sensorless control methods of IMs is mentioned at the beginning of this paper, essentially in terms of MRAS possibilities, with a detailed description of MRAS estimators. In the next chapter, simulation and experimental results of an offline-trained feedforward neural network (ANN-Q-MRAS) estimator are presented in comparison with the conventional Q-MRAS. The results for different speed setpoints and different motor parameters were measured. In conclusion, the suitability of this type of observer is discussed based on the simulation and experimental results.

## 2. Proposed ANN-Based Q-MRAS Estimator

The MRAS estimator examined in the simulations and in the experiment is based on reactive power computation. It belongs to the RP-MRAS category and is related to the P-MRAS, with the primary distinction being whether active or reactive power is used in the computation. One of the significant advantages of this approach is its insensitivity to stator resistance changes, in contrast to the RF- and BEMF-MRAS estimators.

The approach used in this case, as shown in Figure 2, was direct field-oriented control (DFOC), which is the main difference compared with other widely used Q-MRAS methods with indirect field-oriented control (IFOC). The advantage is the closed-loop integration, which is more stable in comparison with IFOC.

### 2.1. Mathematical Model of an Induction Machine

Both observers and open-loop estimators use knowledge of the mathematical model of the relevant electrical machine—in our case, an induction machine. This is expressed in terms of the stator currents and rotor magnetic fluxes of the machine. The description in the stationary reference frame-oriented system [α,β] is then as follows:

(1) $$\frac{d}{dt}\left[\begin{array}{c} i_{s}^{s}\\ \Psi_{R}^{s} \end{array}\right]=\left[\begin{array}{cc} -\frac{L_{m}^{2}R_{R}+L_{R}^{2}R_{s}}{\sigma L_{s}L_{R}^{2}} & \frac{L_{m}R_{R}}{\sigma L_{s}L_{R}^{2}}-\frac{L_{m}}{\sigma L_{s}L_{R}}j\omega_{m}\\ \frac{L_{m}R_{R}}{L_{R}} & -\frac{R_{R}}{L_{R}}+j\omega_{m} \end{array}\right]\left[\begin{array}{c} i_{s}^{s}\\ \Psi_{R}^{s} \end{array}\right]+\left[\begin{array}{c} \frac{1}{\sigma L_{s}}\\ 0 \end{array}\right]u_{s}^{s},$$

(2) $$\sigma =1-\frac{L_{m}^{2}}{L_{S}L_{R}}.$$

where $i_{s}^{s}$ is the stator frame-oriented current, with components $i_{s\alpha }$, $i_{s\beta }$; $u_{s}^{s}$ is the stator frame-oriented voltage, with components $u_{s\alpha }$, $u_{s\beta }$; $\Psi_{R}^{s}$ is the rotor frame-oriented flux, with components $u_{s\alpha }$, $u_{s\beta }$; $L_{m}$ is the mutual inductance; $L_{S}$ and $L_{R}$ are the stator and rotor inductances, respectively; $R_{S}$ and $R_{R}$ are the stator and rotor resistances, respectively; $\omega_{m}$ is the rotor speed; and σ is the leakage factor.

The state description of the mathematical model of the asynchronous machine is defined by the state Equations (3) and (4):

(3) $$\dot{x}=Ax+Bu,$$

(4) y=Cx,

(5) $$x={\left[\begin{array}{cccc} i_{s\alpha } & i_{s\beta } & \psi_{R\alpha } & \psi_{R\beta } \end{array}\right]}^{\;T},\;u={\left[\begin{array}{cc} u_{s\alpha } & u_{s\beta } \end{array}\right]}^{\;T},$$

(6) $$I=\left[\begin{array}{cc} 1 & 0\\ 0 & 1 \end{array}\right],\;J=\left[\begin{array}{cc} 0 & -1\\ 1 & 0 \end{array}\right],\;0=\left[\begin{array}{cc} 0 & 0\\ 0 & 0 \end{array}\right],$$

(7) $$A=\left[\begin{array}{cc} a_{11}I & a_{13}I-a_{14}J\\ a_{31}I & a_{33}I+a_{34}J \end{array}\right]=\left[\begin{array}{cccc} a_{11} & 0 & a_{13} & a_{14}\\ 0 & a_{11} & -a_{14} & a_{13}\\ a_{31} & 0 & a_{33} & -a_{34}\\ 0 & a_{31} & a_{34} & a_{33} \end{array}\right],$$

(8) $$A=\left[\begin{array}{cccc} -\frac{L_{m}^{2}R_{R}+L_{R}^{2}R_{S}}{\sigma L_{S}L_{R}^{2}} & 0 & \frac{L_{m}R_{R}}{\sigma L_{S}L_{R}} & \frac{L_{m}}{\sigma L_{S}L_{R}}\omega_{m}\\ 0 & -\frac{L_{m}^{2}R_{R}+L_{R}^{2}R_{S}}{\sigma L_{S}L_{R}^{2}} & -\frac{L_{m}}{\sigma L_{S}L_{R}}\omega_{R} & \frac{L_{m}R_{R}}{\sigma L_{S}L_{R}^{2}}\\ \frac{L_{m}R_{R}}{L_{R}} & 0 & -\frac{R_{R}}{L_{R}} & -\omega_{m}\\ 0 & \frac{L_{m}R_{R}}{L_{R}} & \omega_{m} & -\frac{R_{R}}{L_{R}} \end{array}\right],$$

(9) $$B=b\left[\begin{array}{c} I\\ 0 \end{array}\right]=\left[\begin{array}{cc} b & 0\\ 0 & b\\ 0 & 0\\ 0 & 0 \end{array}\right]=\frac{1}{\sigma L_{S}}\left[\begin{array}{cc} 1 & 0\\ 0 & 1\\ 0 & 0\\ 0 & 0 \end{array}\right],\;C=\left[\begin{array}{cc} I & 0 \end{array}\right]=\left[\begin{array}{cccc} 1 & 0 & 0 & 0\\ 0 & 1 & 0 & 0 \end{array}\right],$$

where x is the state vector, y is the output vector, A is the state matrix of the induction machine, B is the input matrix, and C is the output matrix.

**Figure 3:**
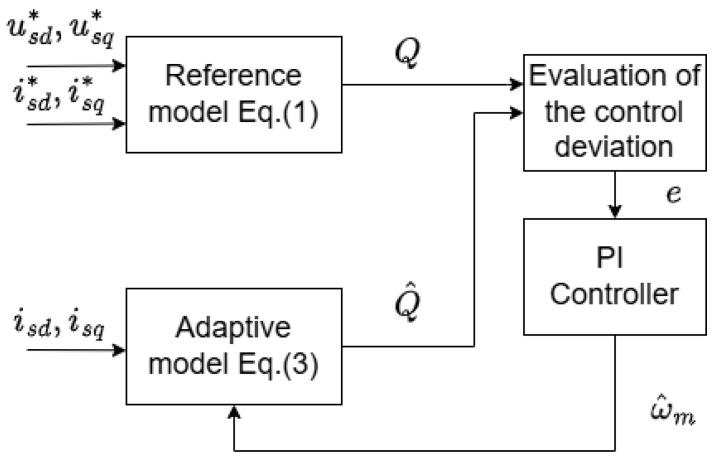
Conventional Q-MRAS estimator.

Figure 3 shows a Q-MRAS that calculates reactive power using quantities measured at motor terminals—this approach is known as terminal reactive power computation. Alternatively, the reactive power can be determined based on values from the air gap.

In the Q-MRAS, where the reactive power is derived from the air gap, a key advantage is its independence from the stator resistance. However, this approach also has drawbacks, including high sensitivity to noise—primarily due to the derivative in the reference model. In addition, it requires accounting for the inductance variation and the estimation of the rotor flux [25,26]. To address these issues, the Q-MRAS was modified to compute the reactive power using the machine’s input parameters instead.

The variation of MRAS used in this work calculates the instantaneous reactive power (IRP), and the equation in the reference model is expressed in Equation (10). For the adaptive model, it is typical to compute reactive power in a steady state (SRP), and the computation is expressed in Equation (12).

(10) $$Q=\vec{i_{S}}\times \vec{u_{S}}=i_{S\alpha }\cdot u_{S\beta }-i_{S\beta }\cdot u_{S\alpha }=i_{Sd}\cdot u_{Sq}-i_{Sq}\cdot u_{Sd}.$$

This implies that the reactive power *Q* is independent of the reference frame, where $i_{S\alpha }$ and $i_{S\beta }$ are the stator currents, $u_{S\alpha }$ and $u_{S\beta }$ are the stator voltages (the reference voltages are used), $i_{Sd}$ and $i_{Sq}$ are the rotor flux-oriented frame currents, and $u_{Sd}$ and $u_{Sq}$ are the rotor flux-oriented frame voltages. The reactive power computed in the adaptive model is conventionally computed in the oriented coordinate system in the following way:

(11) $$\hat{Q}=\sigma L_{S}\cdot {\hat{\omega }}_{m}\left(i_{Sd}^{2}+i_{Sq}^{2}\right)+{\hat{\omega }}_{m}\cdot \frac{L_{m}}{L_{R}}\left(\Psi_{Rq}i_{Sq}+\Psi_{Rd}i_{Sd}\right).$$

For the direct field-oriented control drive, substituting $\Psi_{Rd}=L_{m}i_{Sd}$ and $\Psi_{Rq}=0$ into Equation (11) simplifies the expression to the following form:

(12) $$\hat{Q}=\sigma L_{S}\cdot {\hat{\omega }}_{m}\left(i_{Sd}^{2}+i_{Sq}^{2}\right)+{\hat{\omega }}_{m}\cdot \frac{L_{m}^{2}}{L_{R}}\cdot i_{Sq}^{2}.$$

where $i_{Sd}$ and $i_{Sq}$ are the *d* and *q* components of the current vector of the stator, respectively.

Independence of stator resistance is achieved, and good results are also achieved in low-speed areas, but it is necessary to mention that there are stability problems in the regenerative mode, which are described in [14,22,27]. One of the approaches to improve the stability in the regenerative mode is described in [28], with the so-called multilayer technique. It is also important to know the magnitude of the stator voltage. This can be measured directly, although doing so requires additional voltage sensors, which adds to the cost of the drive. Alternatively, a reference or estimated value can be used instead of the actual measurement. Other motor parameters also influence the estimation, such as the inductance and resistance of the rotor.

The calculation of the reactive power within the reference model is independent of the machine parameters. In contrast, the reactive power estimated by the adaptive model is dependent on the machine parameters, particularly the rotor resistance and leakage inductances.

The adaptation mechanism operates by taking the difference between the reactive powers of the reference and adaptive models, as expressed by the following relation:

(13) $$e=Q-\hat{Q}.$$

The output of the proportional–integral controller is then

(14) $${\hat{\omega }}_{m}=K_{P}\cdot e+K_{I}\cdot \int edt.$$

Within the adaptive model, the estimation of the reactive power accounts for the effects of leakage inductance, which means that the conventional method is sensitive to variations in machine parameters and thereby impacts the estimation accuracy.

The input signals to the proposed Q-MRAS consist of the stator currents and reference voltages expressed in the stationary reference frame, without transformation into a rotating reference frame, which differs from the method outlined in [22].

The calculation of reactive power within the reference model adheres to Equation (10) used in the conventional terminal-based Q-MRAS approach. In the adaptive model, the estimation of reactive power is performed by a neural network-based estimator, which has been trained using the stator current component $i_{Sq}$ (transformed into the field-oriented reference frame) and the measured rotor speed $\omega_{m}$.

In the context of vector control of electric machines, the key parameter used to analyze torque production and optimize control strategies, especially in field-oriented control (FOC) schemes, is the gamma angle $\gamma_{R}$, which represents the angle between the rotor flux vector and the α axis of the stationary reference frame. Accurate determination contributes to efficient torque generation and dynamic performance. The approach to obtain the $\gamma_{R}$ angle is directly from the estimated rotor speed, which means that the current model with a closed integration loop is used.

(15) $${\hat{\Psi }}_{R\alpha \beta }=\int \left[\left(j{\hat{\omega }}_{m}-\frac{1}{\tau_{R}}\right)\Psi_{R_{\alpha }\beta }+\frac{1}{\tau_{R}}L_{m}i_{S\alpha \beta }\right]dt,$$

where ${\hat{\Psi }}_{R\alpha \beta }$ is the rotor flux vector, which is in the same position as the magnetization current vector $i_{m}$; the next variable $i_{S\alpha \beta }$ represents the measured stator current vector in the stationary reference frame [α,β]; and $\tau_{R}$ represents the time constant of the rotor, defined as $\tau_{R}=\frac{L_{R}}{R_{R}}$.

**Figure 4:**
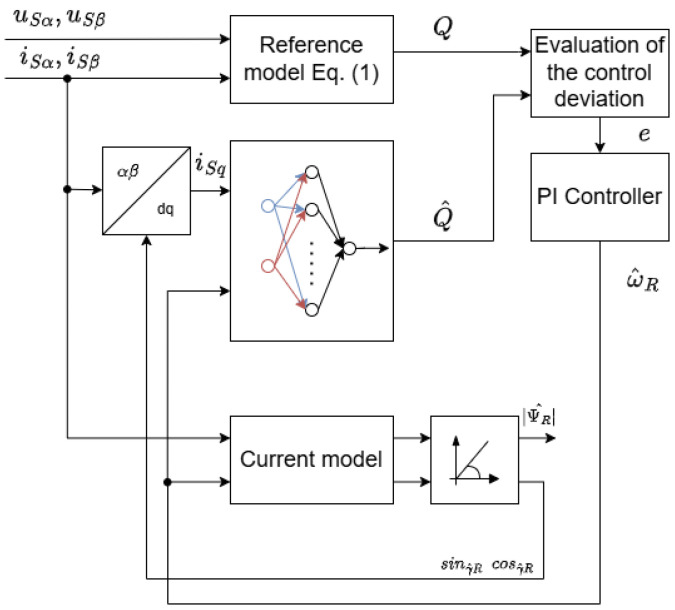
Proposed ANN-based Q-MRAS estimator.

The reactive power *Q* from the reference model was utilized as an output variable. The feedforward neural network features a 2-10-1 architecture. By implementing the network, the dependencies of the IM parameter presented in the adaptive model of the original terminal reactive power Q-MRAS (described in Equation (12)) are eliminated. The block diagram of the ANN-based Q-MRAS is presented in Figure 4. In addition, the number of inputs is reduced to only the torque-producing stator current $i_{Sq}$ and the estimated rotor speed ${\hat{\omega }}_{m}$.

### 2.2. Stability Analysis

Speed estimation algorithms utilizing neural networks have not been extensively examined using classical stability frameworks such as Lyapunov or Popov theory [17]. This study introduces a methodology to evaluate the stability of AI-based MRAS observers, with a focus on the ANN-Q-MRAS algorithm.

Nonlinear autonomous time-invariant systems generally exhibit two types of steady-state behavior: isolated singular points, and limit cycles. The former are discrete equilibrium states in the phase space, whereas the latter represent closed trajectories formed by continuous sets of singular points. Due to the inherent complexity of nonlinear systems, linearization is commonly employed to facilitate analysis. However, such approximations are valid only within constrained operating regions.

The stability of each singular point is assessed by constructing a linear surrogate system through Taylor series expansion around the equilibrium point xs, where f(xs) = 0. Retaining only the linear terms yields a system representation involving the Jacobian matrix and the state-space matrix A (see Equation (16)).

(16) $$J\left(x_{S}\right)={\left[\begin{array}{cccc} \frac{\partial f_{1}\left(x\right)}{\partial x_{1}} & \frac{\partial f_{1}\left(x\right)}{\partial x_{2}} & \ldots & \frac{\partial f_{1}\left(x\right)}{\partial x_{n}}\\ \frac{\partial f_{2}\left(x\right)}{\partial x_{1}} & \frac{\partial f_{2}\left(x\right)}{\partial x_{2}} & \ldots & \frac{\partial f_{2}\left(x\right)}{\partial x_{n}}\\ \vdots & \vdots & \ddots & \vdots \\ \frac{\partial f_{n}\left(x\right)}{\partial x_{1}} & \frac{\partial f_{n}\left(x\right)}{\partial x_{2}} & \ldots & \frac{\partial f_{n}\left(x\right)}{\partial x_{n}} \end{array}\right]}_{x_{S}}.$$

Stability criteria are derived from the classical control theory. For continuous-time systems, asymptotic stability requires that all roots of the characteristic polynomial lie in the left half of the complex plane. For discrete-time systems, stability is ensured if all roots are located within the unit circle. These conditions are consistent with the Lyapunov stability criterion and enable the determination of the poles and zeros of the system [17,29].

The stability of the observer was evaluated using MATLAB Simulink, leveraging functions from the Control System Toolbox to automate the necessary computations. Simulations were conducted in both motor and regenerative modes. The input speed profile followed a triangular waveform [0,45,−45,0,45,−45] over a 6 s period, while the load torque varied according to a repeating sequence [0,−1,−1,1,1,0] with a sampling interval of 1 s. The simulation spanned 60 s, with a fixed time step of $T_{s}=5\cdot 10^{-5}$ s, employing the ode3 (Bogacki–Shampine) solver in accelerator mode.

Linearization points were selected empirically. An initial step size of 0.1 s was used to capture detailed pole-zero behavior. Once the dynamics of the system was sufficiently characterized, the step size was increased to 1 s to improve clarity without compromising analytical integrity.

**Figure 5:**
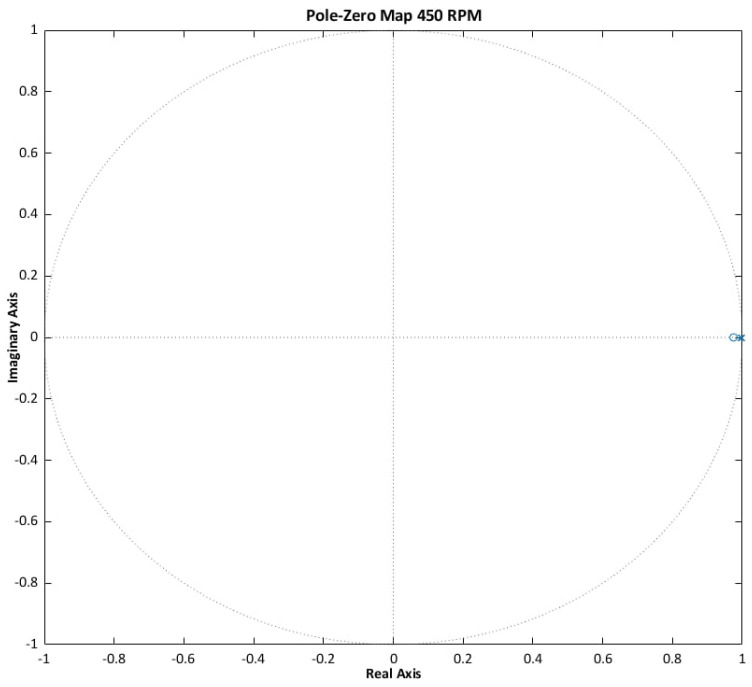
ANN-Q-MRAS pole-zero analysis results.

**Figure 6:**
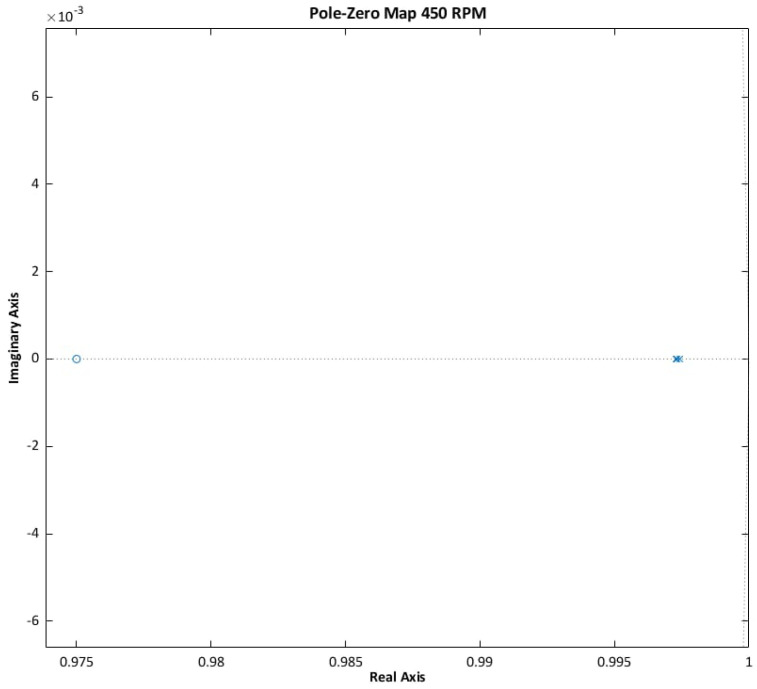
ANN-Q-MRAS pole-zero analysis results zoomed in.

**Figure 7:**
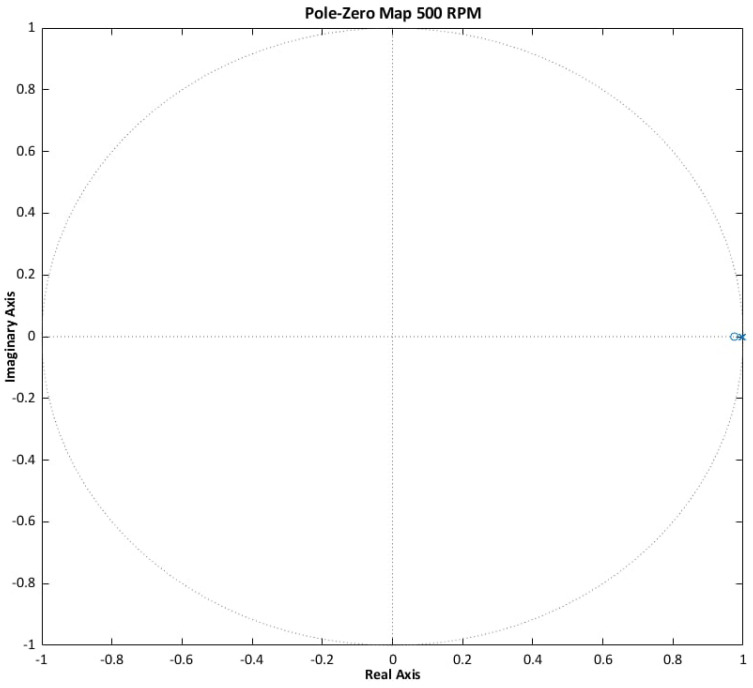
ANN-Q-MRAS pole-zero analysis results for 500 rpm.

The results (see Figure 5, Figure 6 and Figure 7) confirm that the ANN-Q-MRAS algorithm satisfies the established stability criteria. Pole-zero analysis demonstrates robust performance across the evaluated operating conditions.

### 2.3. Implemented Artificial Neural Network

The following section of the paper focuses on the implementation of an artificial neural network for sensorless control of an induction motor drive.

A modified version of the Q-MRAS estimator was developed, and the corresponding simulation and experimental results are presented in Section 3. The neural network was trained in the MATLAB environment using the Neural Network Toolbox, which allows adjustment of the training parameters and network configuration. The training process was performed using the Levenberg–Marquardt algorithm and was run in offline mode. In this approach, training data must be collected in advance and used during the learning phase. After training is complete, the structure and weights of the network remain fixed during drive operation.

**Figure 8:**
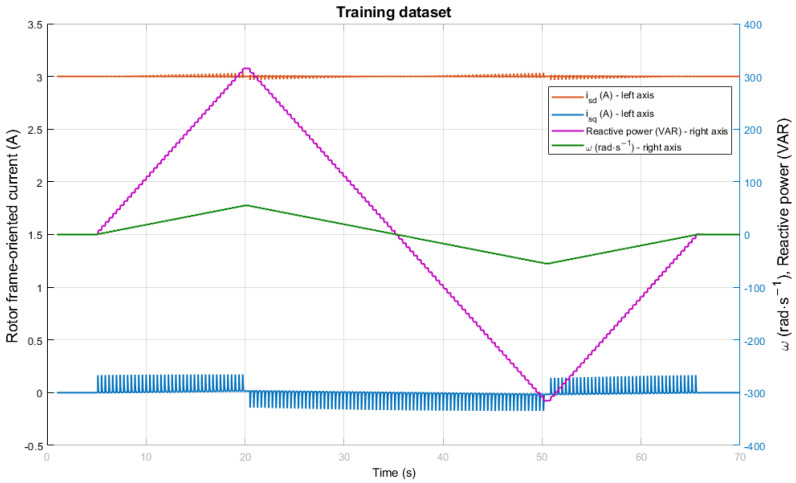
Training dataset for neural network.

A feedforward neural network with a single hidden layer is utilized using the sigmoid activation function. The structure of the used ANN has only two inputs and one output. The number of neurons in the hidden layer is ten; therefore, the network structure is 2-10-1 and was obtained by trial and error. The network receives rotor flux-oriented frame currents, which are obtained from measured stator currents and rotor speed as inputs. The ANN estimates the reactive power of the induction motor as the output. The training data were measured with the DFOC method, with a speed sensor included to obtain the exact rotor speed as one of the inputs for the neural network. The list of used training variables for the network is presented in Table 1. This dataset was used not only for training inputs and outputs but also for further testing of the proper neural network function. The measurement of training data was performed for 70 s using various speeds with no load and a sampling period of 100 μs. Finally, 700,000 patterns were measured, of which 70,000 patterns were randomly selected. The training was terminated in 285 epochs with a Mean Squared Error (MSE) value of 5.61 × $10^{-4}$ VAR. The training data were divided into 65% for network training, 25% for validation, and 10% for network testing. The data collection is shown in Figure 8, where the frame currents oriented to the rotor flux and the measured rotor speed are used as inputs, and the reactive power from the reference model, computed by Equation (10), is used as the output. The left Y axis, which is in amperes, is for the input variables of torque and magnetizing current, while the right Y axis is for the input variable of measured speed in rad · $s^{-1}$, as well as for the output variable of reactive power in VAR. There is no general way to create a neural network, and although several structures and multiple hidden layers were tested, the best performance was achieved with one hidden layer only. This means that the results are highly dependent on the specific application of the network. Therefore, a more complex structure could potentially yield more accurate results. However, the computational power of the used Digital Signal Controller (DSC) is insufficient, as the computation time doubles with each additional network layer.

## 3. Presented Results

The trained neural network with the 2-10-1 structure implemented in the proposed Q-MRAS estimator was validated by simulation in MATLAB Simulink and on an experimental IM laboratory stand. This section presents the simulation and experimental results of the implemented estimator integrated within the direct field-oriented control (DFOC) framework, as shown in Figure 2. For the simulated results, the system was run under no-load conditions. An ANN was trained for this condition as well, but in the experiment, the waveforms were measured with no load and with 20% of the nominal load. The regenerative mode was tested too.

This method excels in low-speed areas, unlike the other MRAS estimators. Implementation of the ANN estimator showed good dynamic response, better stability, and sufficiently accurate estimation in the low-speed range. Based on simulations and experimental results, the sensitivity of the MRAS estimator to changes in machine parameters appeared to be reduced.

### 3.1. Simulation Results

The mathematical model of the induction motor used in the simulations is based on its nominal parameters, as summarized in Table 2. The model incorporates common simplifying assumptions, namely, that mechanical losses, core (iron) losses, and magnetic saturation are neglected. In addition, the control voltage is assumed to be supplied by an ideal power source, with switching effects omitted.

**Figure 9:**
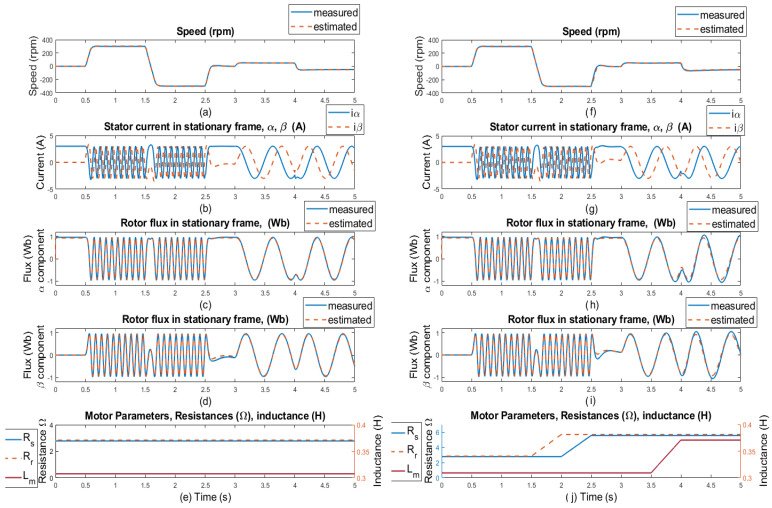
Simulation results: step changes of speed setpoints for **±300 and ±50 rpm, no load, no motor parameters change** (left side of the results); and for **±300 and ±50 rpm, no load, and selected motor parameter changes** ($R_{S}$, $R_{R}$, $L_{m}$-right side of the results). (**a**,**f**) Comparison of measured and estimated speed, (**b**,**g**) stator current in [α, β] coordinates, (**c**,**h**) comparison of measured and estimated α component of the rotor flux, (**d**,**i**) comparison of β components of the rotor flux, and (**e**,**j**) selected motor parameters.

In Figure 9, the simulation results of the rotor speed, stator currents, and rotor flux are presented with selected motor parameters. The waveforms marked as measured (blue lines) were obtained directly from the motor model, and the waveforms marked as estimated (orange lines) were obtained using an ANN-Q-MRAS-based estimator. Figure 9a,f display the simulated responses of the reference and estimated motor speed for speed commands of ±300 rpm and ±50 rpm, panels (b) and (g) in Figure 9 display the stator current waveforms in the stator reference frame [α, β], panels (c) and (h) in Figure 9 display comparisons of measured and estimated α components of the rotor flux, panels (d) and (i) in Figure 9 display β components of measured and estimated components of the rotor flux, and panels (e) and (j) in Figure 9 display the motor parameters under which the system operated. No-load conditions were maintained throughout the entire simulation period. Motor parameters such as the resistances of the rotor and the stator ($R_{s}$ and $R_{R}$, respectively) and the main inductance $L_{m}$ correspond to the real induction motor used for the experimental results.

The changes in the motor parameters can be seen at the time of 1.5 s, where there is a 100% increase in the resistance value of the rotor, from 2.84Ω to 5.68Ω. At the timestamp 2 s, there is a 100% increase in the resistance of the stator, from 2.78Ω to 5.56Ω. The last parameter change is the main inductance $L_{m}$, at the 3.5 s timestamp, which increases by about 20%, from 0.309H to 0.3708H. All parameter changes happen in the 0.5 s window, and estimation is capable of suitable results when all parameters differ at the same time, which confirms that the proposed neural network in the MRAS solution is stable even though applied changes to the system—and, thus, less dependence on motor parameters—were proven.

### 3.2. Experimental Results

**Figure 10:**
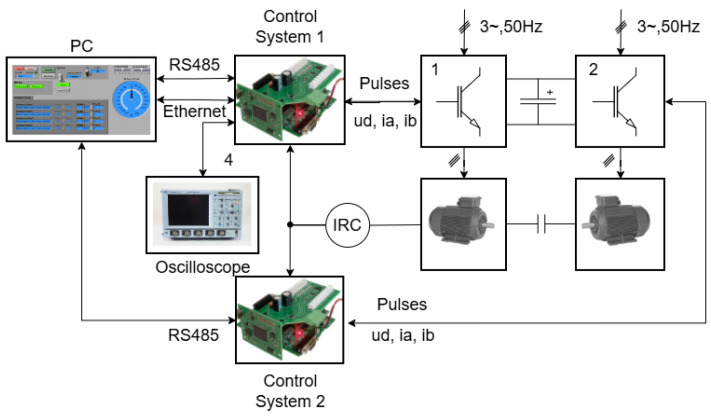
Diagram of the experimental stand.

**Figure 11:**
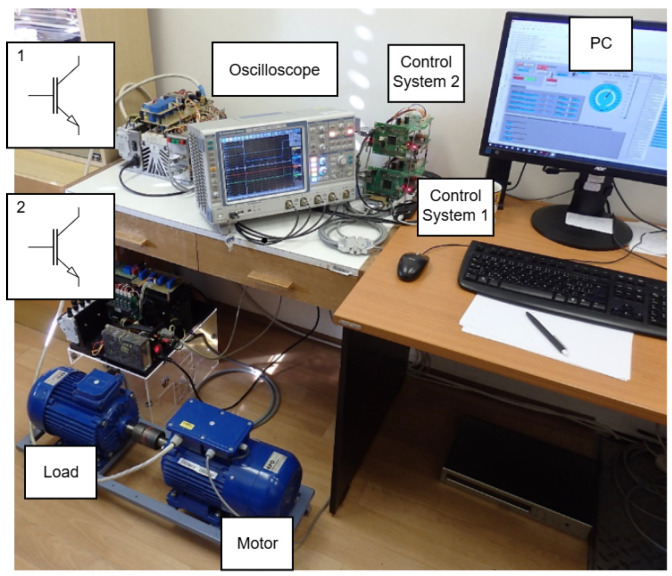
Photo of the experimental stand.

The proposed sensorless control method, including the rotor speed observer and the algorithm with an implemented offline-trained feedforward neural network, was experimentally validated in a laboratory prototype of the induction motor drive with the DFOC structure, as shown in Figure 2. The prototype consists of an Sg 100L-4A induction motor (its parameters are defined in Table 2) fed by an indirect frequency converter with a voltage-source inverter, a second induction motor used for loading, and a control system based on the Texas Instruments Digital Signal Controller TMS320F28335 (DSC). The implemented algorithm of field-oriented control is depicted in Figure 2. The estimation block consists of the presented ANN-Q-MRAS estimator, which can be seen in Figure 4. The block diagram of the proposed system is shown in Figure 10, and a photo of the experimental stand is presented in Figure 11. This floating-point controller is designed for use in embedded real-time systems such as those found in electric drive applications. A sampling frequency of 20 kHz was set for the real-time processing of the algorithms. The rotor speed was measured by an incremental rotary encoder (IRC) with a resolution of 2048 pulses per rotation. The DC-link voltage was set to 200 V for all experimental results. The control system used in this paper was developed in the Department of Applied Electronics of the Technical University of Ostrava. It is intended for control applications in the areas of electric drives and power electronics [30].

**Figure 12:**
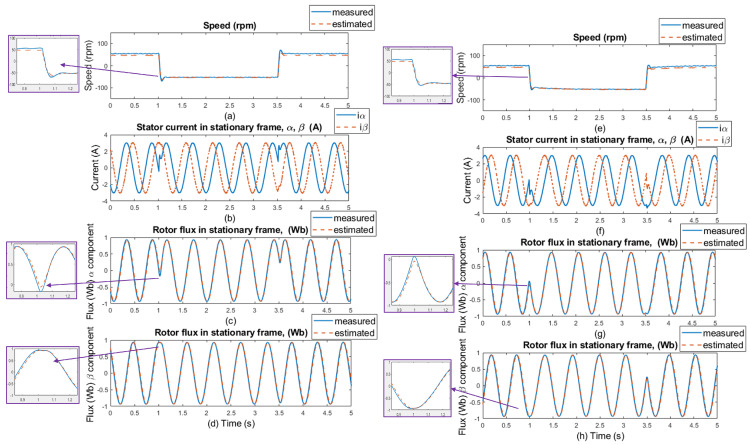
Experimental results: step changes of speed setpoints for **±50 rpm, no load, no motor parameters change** (left side of the results); and with **±50 rpm, no load, selected motor parameter change (20% stator resistance increase)** (right side of the results). (**a**,**e**) Comparison of measured and estimated speed, (**b**,**f**) stator current in [α, β] coordinates, (**c**,**g**) comparison of measured and estimated α components of the rotor flux, and (**d**,**h**) comparison of measured and estimated β components of the rotor flux.

**Figure 13:**
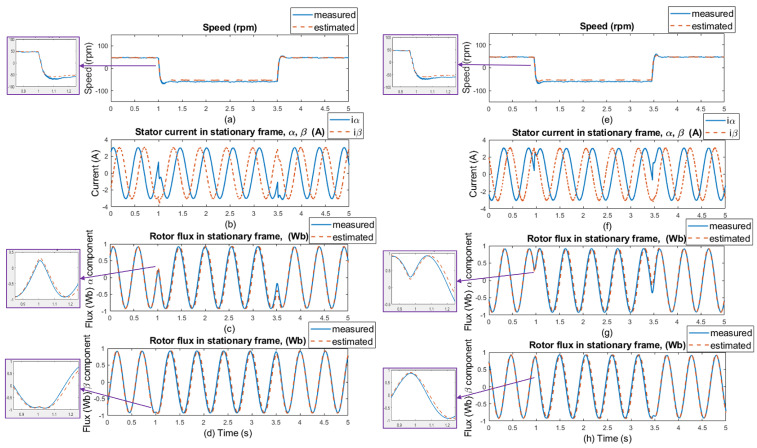
Experimental results: step changes of speed setpoints for **50 rpm (motor mode); −50 rpm (generator mode) load is 20% of nominal torque, no motor parameters change** (left side of the results); and **±50 rpm, load is 20% of nominal torque, selected motor parameter change (20% stator resistance increase)** (right side of the results). (**a**,**e**) Comparison of measured and estimated speed, (**b**,**f**) stator current in [α, β] coordinates, (**c**,**g**) comparison of measured and estimated α components of the rotor flux, and (**d**,**h**) comparison of measured and estimated β components of the rotor flux.

**Figure 14:**
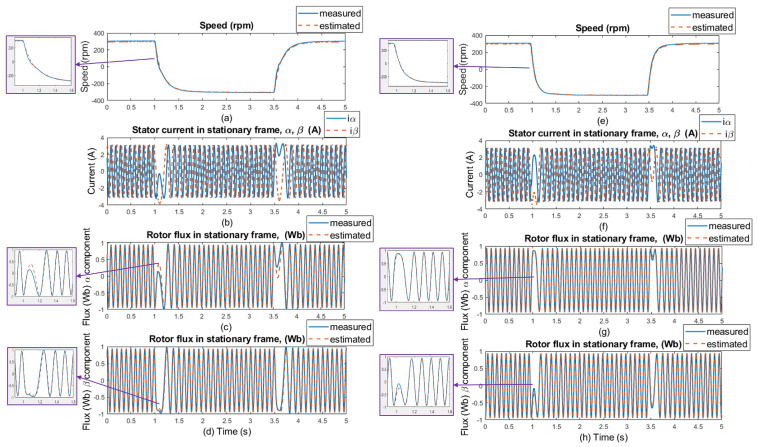
Experimental results: step changes of speed setpoints for **±300 rpm, no load, no motor parameters change** (left side of the results); and for **±300 rpm, no load, selected motor parameter change (20% stator resistance increase)** (right side of the results). (**a**,**e**) Comparison of measured and estimated speed, (**b**,**f**) stator current in [α, β] coordinates, (**c**,**g**) comparison of measured and estimated α components of the rotor flux, and (**d**,**h**) comparison of measured and estimated β components of the rotor flux.

**Figure 15:**
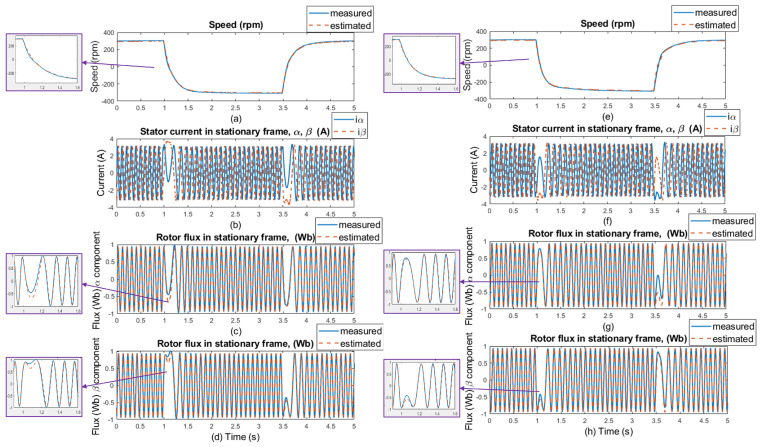
Experimental results: step changes of speed setpoints for **±300 rpm, load is 20% of nominal torque, no motor parameters change** (left side of the results); and for **±300 rpm, load is 20% of nominal torque, selected motor parameter change (20% stator resistance increase)** (right side of the results). (**a**,**e**) Comparison of measured and estimated speed, (**b**,**f**) stator current in [α, β] coordinates, (**c**,**g**) comparison of measured and estimated α components of the rotor flux, and (**d**,**h**) comparison of measured and estimated β components of the rotor flux.

The experimental results were measured in 5 s windows for several speed areas. The measurements were performed without load and with 20% of the nominal load value, with and without motor parameter changes (stator resistance). The regenerative mode (the second quadrant) of the IM drive’s operation is shown in the figures with the load torque. The load was kept constant at 20% of the nominal torque value, which was 3 Nm, while the speed was reversed every after 2.5 s. Figure 12 and Figure 13 show waveforms of the measured and estimated rotor speeds with changes in load and motor parameters for ±50rpm, along with measured currents and measured and estimated rotor flux in the stationary frame. Figure 14 and Figure 15 show the same waveforms for ±300rpm, with and without a load change, and with and without changes in motor parameters.

**Figure 16:**
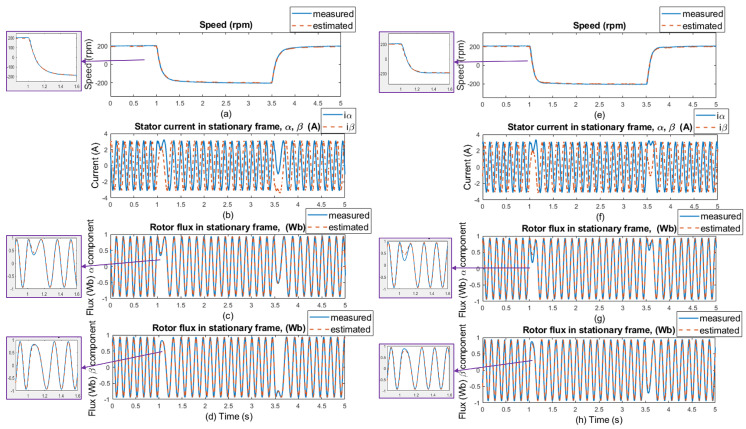
Experimental results: step changes of speed setpoints for **±200 rpm, no load, no motor parameters change** (left side of the results); and for **±200 rpm, no load, selected motor parameter change (20% stator resistance increase)** (right side of the results). (**a**,**e**) Comparison of measured and estimated speed, (**b**,**f**) stator current in [α, β] coordinates, (**c**,**g**) comparison of measured and estimated α components of the rotor flux, and (**d**,**h**) comparison of measured and estimated β components of the rotor flux.

**Figure 17:**
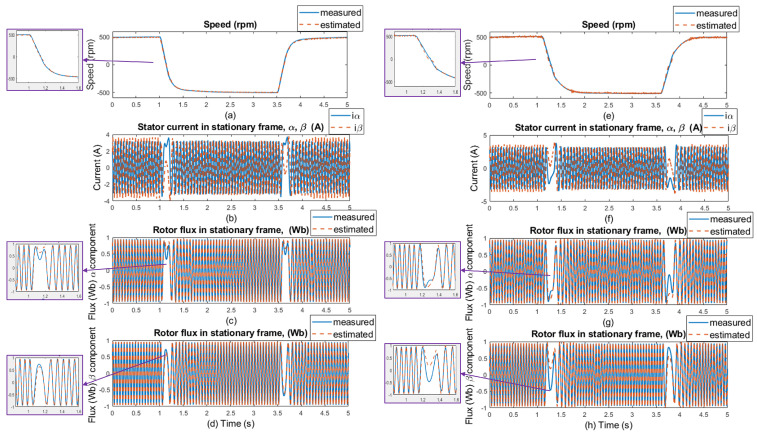
Experimental results: step changes of speed setpoints for **±500 rpm, no load, no motor parameters change** (left side of the results); and for **±500 rpm, no load, selected motor parameter change (20% stator resistance increase)** (right side of the results). (**a**,**e**) Comparison of measured and estimated speed, (**b**,**f**) stator current in [α, β] coordinates, (**c**,**g**) comparison of measured and estimated α components of the rotor flux, and (**d**,**h**) comparison of measured and estimated β components of the rotor flux.

**Figure 18:**
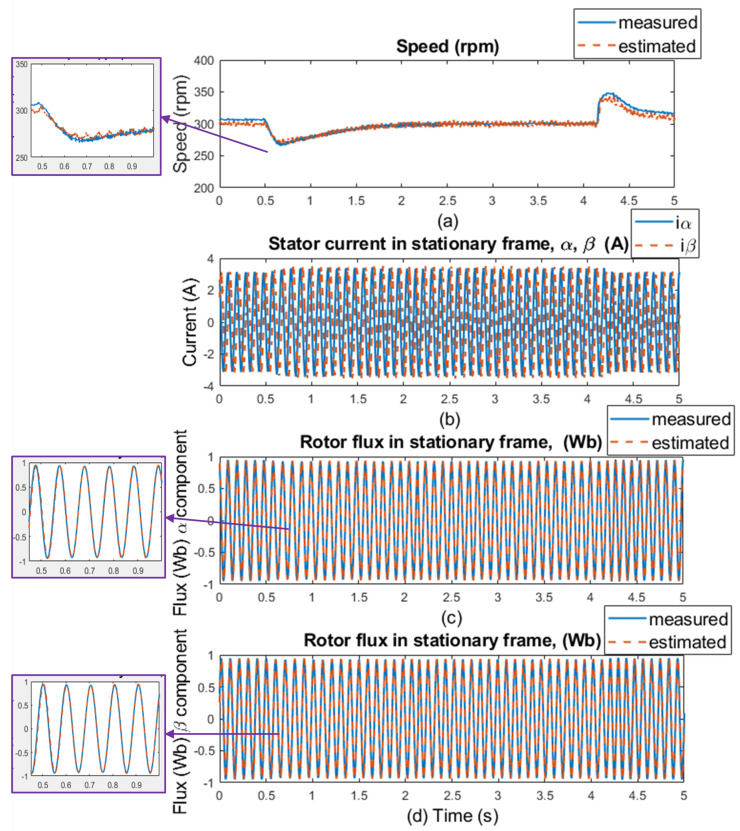
Experimental results: step changes of load for **300 rpm speed, no motor parameters change**. (**a**) Comparison of measured and estimated speed, (**b**) stator current in [α, β] coordinates, (**c**) comparison of measured and estimated α components of the rotor flux, and (**d**) comparison of measured and estimated β components of the rotor flux.

The good dynamics of estimated speeds is shown in the speed comparison section of Figure 12 and Figure 14. It can be said that the estimator works just as well as in sensor mode. Furthermore, in panels (c), (d), (g), and (h) of Figure 12 and Figure 14, a slight phase shift in the measured and estimated rotor flux α and β components in dynamic response can be seen, which is caused by the current model, which uses estimated instead of measured rotor speed to generate the gamma angle. In Figure 13 and Figure 15, when 20% of the nominal load value is applied to the IM, especially in the low-speed area ±50 rpm, there is a visible offset between the measured and estimated speed, specifically in the negative speed range. This is caused by the direction of the load torque, which operates in the same direction as the asynchronous motor; therefore, there is a regenerative mode range that causes this deviation, and the estimator was verified to work in this condition. At higher speeds, this difference is not so noticeable because the load torque does not have such a significant effect. All results show a good response for both the original stator resistance and the modified value of this parameter. Also different speed setpoints was verified as shown in Figure 16 and Figure 17. A sudden load was verified as shown in Figure 18, where the estimator worked properly for the duration of the experiment.

Static and dynamic results of the experiments are summarized in Table 3. The data are divided here according to the speed setpoints, load, and motor parameter changes. The control quality is evaluated using the criteria Integral of Time multiplied by Absolute Error (ITAE) and Mean Squared Error (MSE), which shows slightly better results in the higher speed range; however, even in the lower-speed area, the control quality remains acceptable.

## 4. Conclusions

In this paper, the conventional Q-MRAS technique is introduced, along with its advantages and limitations. The most problematic issues with this estimator are dependency on the IM parameters and instability in the regenerative mode. In the proposed solution, the adaptive model of the Q-MRAS is replaced by an offline-trained feedforward artificial neural network with one hidden layer and a 2-10-1 structure. The performance of the modified ANN-based Q-MRAS estimator was validated through simulations in MATLAB Simulink, and the experimental testing was performed on the IM drive using a control system based on the Texas Instruments Digital Signal Controller TMS320F28335.

The experimental results confirm the simulations, and the suitability of this method was proven in the application. The proposed ANN-based Q-MRAS also operates in the regenerative mode, unlike the original solution. Its performance has been experimentally verified over a wide range of rotational speeds.

The ANN implementation exhibits improved dynamic performance, enhanced stability, and accurate estimation even at low speeds. The simulation and experimental results further indicate a notable reduction in the estimator’s sensitivity to the machine parameter variations.

Future work will focus on testing of the observer in the field-weakening region, and on the implementation of the proposed algorithm on multi-core systems to improve computational efficiency and enable faster real-time processing. Additionally, an online neural network version will be developed and tested, allowing continuous adaptation and learning during operation.

## Figures and Tables

**Table 1:** Training data for neural network.

Symbol (Unit)	Description
$i_{Sd}$, $i_{Sq}$ (A)	Measured rotor flux-oriented frame currents
$\omega_{m}$ (rad · $s^{-1}$)	Measured rotor speed
Q (VAR)	Reactive power

**Table 2:** Nominal parameters of the IM Sg 100L-4A.

Parameter	Value	Parameter	Value
Power	2.2 kW	$R_{S}$	2.78 Ω
Rotor Speed	1425 rpm	$R_{R}$	2.84 Ω
Torque	14.7 Nm	$L_{S}$	318.9 mH
Voltage (Y/D)	400/230 V	$L_{R}$	318.1 mH
Current (Y/D)	4.8/8.8 A	$L_{m}$	309 mH
Frequency	50 Hz	$R_{\mathrm{Fe}}$	1667 Ω
Cos φ	0.8	p	2
Moment of Inertia	0.0065 ${\mathrm{kgm}}^{2}$	$\tau_{R}$	112 ms

**Table 3:** Summary of the measured experimental results.

	Experiment	Parameters			
Figure	Speed (rpm)	Load (%)	Stator Resistance Change (%)	ITAE Error (rpm)	MSE Error (rpm)
Figure 12a–d	50	0	0	$8.9\times 10^{-7}$	$5.59\times 10^{-2}$
Figure 12e–h	50	0	20	$8.61\times 10^{-7}$	$5.9\times 10^{-2}$
Figure 13a–d	50	20	0	$4.49\times 10^{-7}$	$2.09\times 10^{-2}$
Figure 13e–h	50	20	20	$5.74\times 10^{-7}$	$1.18\times 10^{-2}$
Figure 14a–d	300	0	0	$3.88\times 10^{-7}$	$1.23\times 10^{-2}$
Figure 14e–h	300	0	20	$4.69\times 10^{-7}$	$2.2\times 10^{-2}$
Figure 15a–d	300	20	0	$5.03\times 10^{-7}$	$1.81\times 10^{-2}$
Figure 15e–h	300	20	20	$4.59\times 10^{-7}$	$1.23\times 10^{-2}$
Figure 16a–d	200	0	0	$3.65\times 10^{-7}$	$2.75\times 10^{-2}$
Figure 16e–h	200	0	20	$4.4\times 10^{-7}$	$2.25\times 10^{-2}$
Figure 17a–d	500	0	0	$3.29\times 10^{-7}$	$1.27\times 10^{-2}$
Figure 17e–h	500	0	20	$2.11\times 10^{-7}$	$0.44\times 10^{-2}$

## Data Availability

The original contributions presented in this study are included in the article. Further inquiries can be directed to the corresponding author.

## Abbreviations

The following abbreviations are used in this manuscript:

AC	Alternating current
ANN	Artificial neural network
ANN-MRAS	Artificial intelligence-based MRAS
ANN-Q-MRAS	Artificial neural network reactive power-based MRAS
BEMF-MRAS	Back electromotive force-based MRAS
CB-MRAS	Current-based MRAS
DC	Direct current
DFOC	Direct field-oriented control
DSC	Digital Signal Controller
FL-MRAs	Fuzzy logic-based MRAS
IFOC	Indirect field-oriented control
IM	Induction machine
IRC	Incremental rotary encoder
IRP	Instantaneous reactive power
ITAE	Integral of time-weighted absolute error
MSE	Mean Squared Error
MRAS	Model reference adaptive system
P-MRAS	Power-based MRAS
Q-MRAS	Reactive power-based MRAS
RF-MRAS	Rotor flux-based MRAS
SM-MRAS	Sliding mode-based algorithm MRAS
SRP	Steady-state reactive power
VAR	Volt-ampere reactive
X-MRAS	Virtual variable-based MRAS
Y-MRAS	Virtual variable-based MRAS
